# Detection of Procedural Errors with Stainless Steel and NiTi Instruments by Undergraduate Students Using Conventional Radiograph and Cone Beam Computed Tomography

**Published:** 2013-10-07

**Authors:** Regis Augusto Aleixo Alves, João Batista Souza, Ana Helena Gonçalves Alencar, Jesus Djalma Pécora, Carlos Estrela

**Affiliations:** a Department of Stomatologic Science, Dental School, Federal University of Goiás, Goiânia, GO, Brazil;; b Department of Conservative Dentistry, Dental School, Federal University of Goiás, Goiânia, GO, Brazil;; c Department of Conservative Dentistry, Dental School, University of São Paulo, SP, Brazil

**Keywords:** Cone Beam Computed Tomography, Dental Education, Dental Instruments, Nickel-Titanium, Perforation, Root Canal Preparation

## Abstract

**Introduction:**

This study investigated procedural errors made during root canal preparation using stainless steel and nickel-titanium (NiTi) instruments by undergraduate students, using two diagnostic imaging methods.

**Materials and Methods:**

Sixty human molars were divided into three groups (*n*=20; group 1: K-Flexofile, group 2: K3, and group 3: BioRace). The root canals were filled with gutta-percha and AH Plus. Periapical radiographs and cone beam computed tomography (CBCT) images were obtained to detect procedural errors made by undergraduate students during root canal preparation. Two examiners evaluated the presence or absence of fractured instruments, perforations and canal transportations. The agreement between observers was assessed using the kappa coefficient. The Kolmogorov-Smirnov, Fisher exact, ANOVA and Tukey tests were used for statistical analysis. The level of significance was set at 5%.

**Results:**

There were no significant differences in detecting procedural errors between two- and three-dimensional diagnostic imaging methods. There were no significant differences in procedural errors between stainless steel and NiTi instruments. Mean preparation time was recorded in minutes, and results were significantly different between the three groups. NiTi instruments had the lowest mean preparation time.

**Conclusion:**

Both periapical radiographs and CBCT identified procedural errors, however, three-dimensional images offered more diagnostic resources. The frequency of procedural errors was low for any of the endodontic instruments despite being used by inexperienced operators.

## Introduction

Contemporary endodontics has undergone important changes with the development of new methods and instruments. The introduction of nickel-titanium (NiTi) for orthodontic archwires, favored the production of new instruments in endodontics [1]. Walia, *et al*. studied the properties of this alloy in endodontic instruments, and their results gained the attention of the industry [2]. The superelasticity of NiTi enabled the preparation of curved root canals with better quality.

During last years, the use of engine-driven NiTi instruments for the preparation of curved root canals has been gradually incorporated into the curriculum of undergraduate courses. Endodontic courses for under-graduate students should include updated scientific knowledge which covers comprehensive methodological strategies as well as science of materials [3-6].

Instrumentation of curved root canals is one of the critical procedures of endodontic therapy [7]. The flexibility of NiTi instruments preserves the original shape of the root canal and ensures a better canal curvature compared to stainless steel instruments (K-flex) [8].

Several factors favor the adoption of NiTi rotary instruments for root canal preparation including a more centralized preparation, maintenance of the working length, fewer procedural errors and better quality [2, 3, 8-10]. New NiTi rotary instruments with different characteristics (cross-section, cutting angle, helical angle, radial grooves/edge, flutes, *etc.*) have been introduced into the profession [1, 9, 10].

Numerous dental schools have included the use of rotary instrumentation in their curriculum. However, there is still strong resistance against it. Several studies showed that undergraduate students make few procedural errors when using NiTi rotary instruments [3, 4, 11-13]. Moreover, the cost of NiTi instruments is rather low, and teaching this technique in graduate programs seems time consuming [11]. Spangberg [14] reported that although the rotary technique is not a basic procedure for undergraduate training, general practitioners and endodontists use these instruments. Therefore, it was logical and natural for schools of dentistry to teach at least one technique of using NiTi rotary instruments. Pécora *et al*. [5] emphasized the importance of using these mechanized systems in NiTi endodontics, as well as their application in laboratory and clinical activities during the undergraduate course.

All factors that may influence the inclusion of these resources and knowledge in the undergraduate curriculum, such as the risk of instrument fracture, root canal perforation and apical transportation, should be evaluated together with the need for preclinical training. Therefore, based on the performance of NiTi rotary instruments in the preparation of curved root canals, several studies evaluated the use of these resources in undergraduate teaching using various methods [3, 9-12].

Parallel with all advances in dentistry technologies, cone beam computed tomography (CBCT) has been used for different purposes in endodontics, such as study of root canal anatomy, three-dimensional simulation of internal and external tooth structures, evaluation of root canal preparation, obturation, retreatment, diagnosis and treatment of bone lesions [15-24]. Its ability to reduce or eliminate the superimposition of surrounding structures makes CBCT superior to conventional periapical films [22]. Compared with medical tomography, CBCT has some advantages: lower radiation dose, higher scanning resolution and more accuracy of volume measuring in different directions due to voxels being isotropic which make them different from CT [16].

Based on the importance of introducing modern root instrumentation techniques and new imaging resources into undergraduate courses, this study evaluated procedural errors made by undergraduate students during root canal preparation with stainless steel and NiTi instruments using periapical radiography and CBCT.

## Material and Methods

This study was approved by the Ethics Committee of the Federal University of Goiás (Proc. # 042/2011), Brazil, and an informed consent form was obtained from all patients.

### Selection and tooth preparation

Sixty extracted human maxillary and mandibular molars were obtained from the Dental Urgency Service of the School of Dentistry in Federal University of Goiás, Brazil. The teeth were stored in 0.2% thymol solution and then immersed in 5% NaOCl for 30 min to remove external organic tissues.

### Image acquisition

Preoperative radiographs of each tooth were taken to confirm the absence of calcified root canals, internal/external root resorption, and the presence of a fully formed apex. Radiographic images were acquired using a Spectro X70 electronic X-Ray unit (Dabi Atlante, Ribeirão Preto, Brazil), 0.8×0.8 mm tube focal spot, Kodak Insight film-E (Eastman Kodak Co, Rochester, NY, USA) and paralleling technique. A radiographic platform was used to standardize all radiographs. All films were processed in an automatic processor, and images were evaluated in a dark room using a light box under a magnifying glass.

CBCT images were obtained using an I-CAT Cone Beam 3D imaging system (Imaging Sciences International, Hatfield, PA), with 0.20×0.20×0.20 mm voxel size and 14 bits. Images were examined using the Xoran 3.1.62 software (Xoran Technologies, Ann Arbor, MI) in a PC workstation running Microsoft Windows XP professional SP-2 (Microsoft Corp, Redmond, WA, USA).

### Inclusion and exclusion criteria

Only three-canalled teeth were used in the study (maxillary molars with palatal, mesiobuccal and distobuccal canals and mandibular molars with distal, mesiobuccal and mesiolingual canals). All teeth were shorter than 22 mm, and at least one of the buccal canals of maxillary molars and the mesial canals of mandibular molars had a moderate curvature (4<radius≤8 mm). The root curvature radius (r) was estimated by methods described by Estrela *et al*. [18].

### Standard access cavities

After taking periapical radiographs and CBCT images, standard access cavities were made by an endodontist using round diamond burs (#1013, #1014; KG Sorensen, Barueri, SP, Brazil) and Endo Z burs (Dentsply-Maillefer, Ballaigues, Switzerland), with a high speed handpiece and air-water spray cooling.

### Working length determination

The working length (WL) was determined using #10 and #15 K-Flexofiles (Dentsply, Ballaigues, Switzerland) which were introduced into the root canals until being visible at the apical foramen. The WL was set 1 mm short of the apex.

### Student selection

To evaluate the root canal preparation by inexpert operators, undergraduate students of the School of Dentistry of Federal University of Goiás were invited to participate in the study. Each student prepared/filed 30 root canals of each group. They did not have any experience in the preparation of curved root canals, and had an 8-h theoretical course on rotary instrumentation associated with clinical applications.

### Group assignment

The root canals were randomly divided into three experimental groups of 20 teeth each (n=60 canals) and prepared using the following instruments: *G1*- stainless steel files (K-Flexofile, Dentsply-Maillefer, Switzerland); *G2*- K3 (SybronEndo, Orange, CA); and *G3*- BioRace (FKG Dentaire, La Chaux-de-Fonds, Switzerland).

### Root canal preparation

Gates Glidden drills sizes #1 and 2 were used in *G1*, files #25/.10 and #25/.08 in *G2*, and #25/0.08 BR0 in *G3* for preparation of coronal part of root canals. After working length determination, the apical third was prepared using files #15 to 40 in *G1*. Files up to #35 were used in mesial canals of mandibular molars and buccal canals of maxillary molars; and size #40, was the final apical preparation for the distal canals of mandibular molars and palatal canals of maxillary molars. In *G2*, the sequence used was #15/.02-45/.02, #25/.04 and #25/.06 for all root canals [25]. In *G3*, BR1 (#15/0.05), BR2 (#25/0.04), BR3 (#25/0.06), BR4 (#35/0.04) and BR5 (#40/0.04) were used. For wider root canals of this group (distal canal of mandibular molars and palatal canal of maxillary molars), the BR6 (#50/0.04) and BR7 (#60/0.02) instruments were also used. The root canals were shaped at a rotational speed of 500 rpm (X-Smart, Dentsply, Ballaigues, Switzerland) and 1.5 Ncm torque. Two sets of instruments were used, and the time required for each preparation was recorded using a digital stopwatch.

During preparations, the canals were irrigated between instruments with 3 mL of a recently prepared 1% NaOCl using a syringe with a 30-gauge needle (Injecta, Diadema, Brazil). Root canals were dried and filled with 17% EDTA (pH 7.2; Biodinâmica, Ibiporã, Brazil) for 3 min to remove the smear layer. Another 3 mL of 1% sodium hypochlorite solution was used for irrigation. Periapical radiographs and CBCT images were then taken.

### Root canal filling

The root canals were filled with gutta-percha and AH Plus sealer (Dentsply, Ballaigues, Switzerland) using the conventional lateral condensation technique. The sealer was prepared according to manufacturer’s directions. After obturation, new periapical radiographs and CBCT images were taken under the same conditions described previously.

### Image evaluation

Two examiners (a radiologist and an endodontist) were calibrated using 20% of the specimens, and all images were evaluated to detect the presence or absence of fractured instruments, root perforations (coronal, middle or apical third) and deviations from the original trajectory of the root canal (apical transportation). Instrument fractures during preparation were also detected. When a consensus was not reached by the two examiners that interpreted the procedural errors using the images, a third observer (an endodontist) made the final decision. 

### Statistical Analysis

The agreement between examiners was analyzed using kappa statistics. The differences between types of procedural errors according to periapical radiographs and CBCT images were analyzed using the Kolmogorov-Smirnov test. The association of periapical radiographs and CBCT images with types of procedural errors was evaluated using the Fisher exact test. The differences of mean time for root canal preparation between the three groups were assessed using ANOVA, and the comparison of means, using the Tukey test. The level of significance was set at 5%.

## Results

The results of kappa statistics (κ=0.889) revealed that agreement was significant (*P*<0.001).The frequency of procedural errors detected using periapical radiographs and CBCT images and the mean time of root canal preparation according to instrument used are described in Tables 1-3. In a total of 180 root canals prepared (maxillary and mandibular molars), 11 (6.11%) procedural errors were detected using periapical radiographs (7 [3.88%] fractures and 4 [2.23%] canal transportations). CBCT also detected 11 (6.11%) procedural errors (7 [3.88%] fractures, 3 [1.67%] canal transportations and 1 [0.56%] perforation). 

The analysis of types of procedural errors, instruments and diagnostic imaging methods revealed no significant differences (*P*>0.05). The analysis of time to prepare root canals using different instrumentation systems revealed significant differences between K-Flexofile and K3 (*P*=0.002), K-Flexofile and BioRace (*P*<0.001) and K3 and BioRace (*P*=0.003).

**Figure 1.**
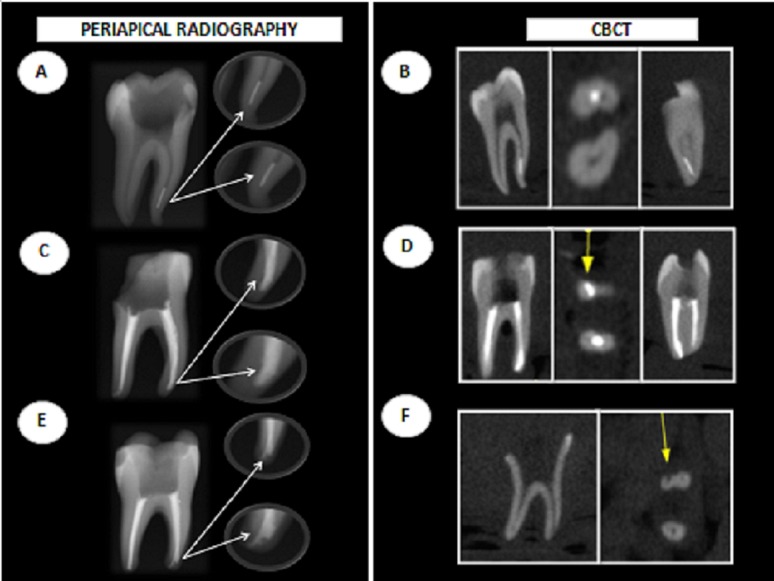
Procedural errors detected using periapical radiographs and CBCT images: instrument fracture (A, B), canal transportation (C, E, D) and perforation (F)

Figure 1 illustrates cases of instrument fracture, canal transportation and perforation detected using periapical radiography and CBCT. 

## Discussion

Probable procedural errors affecting treatment prognosis should be considered and evaluated before choosing a new endodontic instruments to be used. This study found no significant difference in the ability of different imaging techniques in diagnosis of procedural errors, either the occurrence of procedural errors using different canal preparation techniques. [3, 4, 9-11, 13, 26-29].

A total Of 180 root canals were prepared in this study and 11 (6.11%) procedural errors were detected using periapical radiography and CBCT. The quality of the preparation of curved root canals has been assessed using different methods on extracted teeth, such as tooth clearing[30], radiography [3, 8, 9], microcomputed tomography (µ-CT) [9, 21, 28] and CBCT [3, 17, 23] or by using simulated canals [10, 27, 31]. The unpredictability of internal dental anatomy is a great challenge in the preparation of the whole root canal system [31], and studies about noninvasive diagnostic methods should be conducted to detect procedural errors, which are risk factors for endodontic failures [3].

**Table 1.** Frequency of procedural errors in canals detected using periapical radiographs and CBCT (Kolmogorov-Smirnov test).

Method	n	Fracture	Canal Transportation	Perforation	*P*-value
**Periapical radiograph**	180	7 (3.88%)	4 (2.23%)	0 (0.00%)	*P*>0.05
**CBCT[Table-fn fn5381]** ^**a**^	180	7 (3.88%)	3 (1.67%)	1 (0.56%)	*P*>0.05

a. Cone beam computed tomography

**Table 2.** Frequency of procedural errors in canals detected using periapical radiographs and CBCT images (according to instrument).

Method	n	K-Flexofile	K3	BioRace	*P*-value
**Periapical radiograph**	180	3 (27.27%)	3 (27.27%)	5 (45.45%)	*P*>0.05
**CBCT[Table-fn fn5382]** ^**a**^	180	3 (27.27%)	3 (27.27%)	5 (45.45%)	*P*>0.05

a. Cone beam computed tomography

**Table 3.** Mean time of root canal preparation in minutes per tooth according to instrument (ANOVA, Tukey test)

Mean time (SD)	K-Flexofile	K3	BioRace	*P*-value
**Students**	43 (15)	30 (11)	17 (6)	*P*<0.001

Rather resolution of CBCT images and three-dimensional imaging techniques have contributed to the analysis of the internal morphology and are important resources to assess the shape of the root canal before and after preparation [3, 9, 16, 21, 23, 28]. Peters *et al*. [28] evaluated the potential and accuracy of a three-dimensional *in vitro* technique (µ-CT) to describe the geometry of root canals using extracted human molars. They accurately determined the internal anatomy of canals using this innovative technique, and their variables and indices may serve as a basis for studies of root canal anatomy, but this imaging method is not used in clinical situations due to high lethal amount of exposure.

CBCT must be carefully used, especially considering its appropriate indication and method of analysis [15]. Metal artifacts and filling materials may interfere with CBCT images, and, therefore, a periapical radiograph should be obtained in advance for simultaneous analysis with CBCT. Precautions must be taken to deal with the effect of solid materials in the interior space of root canals on CBCT images [15]. To minimize this effect, CBCT images were taken at three time points in this study: before and after instrumentation and after root canal filling. In this study the perforations were detected at the second time point, which ruled out the possibility of a false negative data due to an artifact because the tooth had not been filled yet. Periapical radiographs were also taken at three time points because, within the limitations of this type of imaging, canal transportation is best viewed when the root canals are filled.

The instruments used in this study were stainless steel K-Flexofiles for hand use, the K3 NiTi rotary system and BioRace. The samples were carefully selected and comprised teeth with moderate canal curvatures in at least one of the mesial roots of mandibular molars and buccal roots of maxillary molars (4<r≤8 mm). The occurrence of procedural errors was low regardless of type of instrument used. The results of this study confirm the low frequency of procedural errors during root canal preparation using NiTi instruments [3, 9, 12].

The frequency of errors according to instrument used was not significant. The analysis of type of error revealed that no fracture occurred with the use of stainless steel K-Flexofiles (Tables 1 and 2). The main procedural error detected when using this type of instrument was canal transportation, which may be explained by the fact that stainless steel instruments do not have characteristics of superelasticity or shape-memory effect and tend to straighten the canal. Because of that, operators should pre-curve the file manually and file the root canal walls with short length movements, a technique that is difficult to standardize and this fact favors the occurrence of canal transportation. 

Oliveira *et al*. used CBCT to evaluate apical transportation after root canal preparation using different automated systems (K-Flexofile, Nitiflex, K3 and Race) [23]. Centralizing ability and apical transportation were not influenced by mechanical motion or type of instrument used. Hartmann *et al*. [21] used computed tomography (CT) to compare transportation in the mesiobuccal canals of maxillary molars prepared using different techniques: manual instrumentation with K-Flexofile, K-Flexofile attached to an oscillatory system and ProTaper rotary system. All techniques caused root canal transportation and the oscillatory technique had the greatest reduction from the dentinal walls of inner curvature. Alencar *et al*. [3] compared students in their final undergraduate year and endodontists with over five years of experience to evaluate the occurrence of procedural errors (fracture, perforation and canal transportation) using the ProTaper Universal and found that both undergraduate students and dentists used rotary NiTi instruments successfully and achieved low rates of procedural errors. 

The instrument fractures detected in this study occurred with the use of rotary NiTi instruments, with no significant differences between groups (Table 2). The fracture of NiTi instruments may be associated with the following factors: knowledge, experience, technique, design characteristics and surface treatment [13]. Lopes *et al*. evaluated the effect of electro-polishing as a surface treatment on the number of *cycles to fracture *when using a BioRace instrument (# BR5C) [31]. They concluded that the number of cycles to instrument fracture after electrolyte treatment was 124% higher than the instruments without receiving any surface treatment. 

In this study, mean preparation time was recorded, and results were significantly different between the three groups (Table 3). The lowest mean preparation time belonged to NiTi instruments, that can be justified by the automation of rotary systems, which favors a faster canal preparation. Tu *et al*. [28] prepared 46 simulated curved canals in resin blocks using manual and automated instrumentation with the ProTaper system and found that the learning curve for students is lower for the rotary system than for the manual system. 

Devotion of extra time for preparation of curved canals using NiTi rotary systems may represent a greater risk of fracture. Mesgouez *et al*. determined the influence of operator experience (with and without previous knowledge) at the time of preparation of simulated curved canals using the Profile system [10]. Mean preparation time for all specimens was 2 minutes and 42 seconds per canal. The time required for preparation of the root canal was found inversely proportional to the operator's experience. Sonntag *et al*. [13] stated that operators with little experience performed better root canal preparation when using NiTi rotary instruments compared to stainless steel instruments, although more fractures occurred. Students prepared curved canals at least 2.5 minutes faster, provided that they had preliminary experience with a hand preparation technique.

Gekelman *et al*. evaluated the canals prepared by inexperienced clinicians who had received training sessions [9]. Computer software was used to analyze the canals and assess changes (volume, surface, shape, and transportation) during preparation. The results showed no significant differences between the instruments or operators in relation to variations in the center of mass; qualitative and quantitative data of canal transportation were similar for inexperienced students and experienced operators. Both systems were satisfactory when operated by inexperienced students who received a brief training session.

Dental schools worldwide have assessed the use of rotary NiTi instruments for curved canal preparation [3, 5, 9, 11, 13, 29]. Further research should investigate new concepts and technologies that raise opportunities for discussion, reflection and changes in the scientific world.

## Conclusion

The frequency of procedural errors (fracture, canal transportation and perforation) during the preparation of canals of maxillary and mandibular molars using stainless steel and NiTi instruments was low regardless of diagnostic imaging method when used by inexperienced operators.
